# Young adults looking back at their experiences of treatment and care for nonsuicidal self-injury during adolescence: a qualitative study

**DOI:** 10.1186/s13034-024-00706-2

**Published:** 2024-01-20

**Authors:** H Andersson, E Svensson, A Magnusson, R Holmqvist, M Zetterqvist

**Affiliations:** 1 Center for Social and Affective Neuroscience, Department of Biomedical and Clinical Sciences, Linköping university, Linköping, Sweden; 2 Department of Behavioural Sciences and Learning, Linköping university, Linköping, Sweden; 3 Department of Child and Adolescent Psychiatry, Region Östergötland, Linköping, Sweden

**Keywords:** Nonsuicidal self-injury, Child and adolescent psychiatry, Lived experience, Thematic analysis, Health care professionals, Young adults, Adolescence

## Abstract

**Background:**

Nonsuicidal self-injury (NSSI) is associated with stigma, and negative attitudes among healthcare professionals toward NSSI have been reported. A person-centered approach that focuses on how individuals with lived experience of NSSI perceive the treatment and care they receive is invaluable in reducing barriers to help-seeking and improving treatment and mental healthcare services. The aim of the current qualitative study was to explore the perceptions of young adults when they look back upon their experiences of psychiatric treatment for NSSI during adolescence.

**Methods:**

Twenty-six individuals with lived experience of NSSI who were in contact with child and adolescent psychiatry during adolescence were interviewed. The interviews were analyzed using thematic analysis.

**Results:**

Three main themes were developed: Changed perceptions in retrospect, The importance of a collaborative conceptualization and Lasting impression of the relationship. Participants’ perception of themselves as well as the treatment changed over time. The importance of a joint understanding of NSSI and an agreed-upon treatment focus was emphasized. The relationship to the mental health professionals, and experiences of how NSSI was communicated, were salient several years later.

**Conclusions:**

Healthcare professionals need to communicate about NSSI in a respectful manner and include the perspective of the adolescent with lived experience of NSSI in a joint conceptualization of NSSI and treatment focus.

## Introduction

Nonsuicidal self-injury (NSSI), i.e., deliberately hurting oneself through cutting, hitting or burning body tissue without suicidal intent, is common among adolescents [1–3]. Prevalence rates of about 17% have been found in community samples [4], while 40–80% of adolescents in clinical samples report NSSI [5]. NSSI is generally viewed as a transdiagnostic phenomenon and in clinical samples it is associated with several different conditions, such as depression and anxiety, for example [6]. It is also a symptom of borderline personality disorder, and NSSI with early onset and longer duration in adolescence has shown to predict emergent borderline personality disorder in young adults [7]. Emotion dysregulation has consistently been found to be associated with NSSI [8]. NSSI has been conceptualized as a coping strategy and examples of reasons to engage in NSSI are to cope with difficult emotions and thoughts [9, 10].

Despite progress in research and clinical care of NSSI during the last decade, with the development of clinical guidelines and knowledge dissemination to stakeholders, healthcare professionals, parents, and school staff to raise awareness about NSSI (e.g., [11]), NSSI is still associated with stigma, myths, and misconceptions [12, 13]. Earlier work has shown that healthcare professionals tend to report negative attitudes toward individuals with NSSI [14]. Modern evidence-based treatments that address NSSI therefore emphasize strategies for decreasing iatrogenic effects.

There are treatments that specifically target NSSI in adolescents, and the research field is rapidly evolving [15, 16]. Some examples of treatments are Dialectical Behavioral Therapy for Adolescents (DBT-A; [17]), Emotion Regulation Therapy for Adolescents (ERITA; [18, 19]) and the online STAR program [20]. A qualitative study showed that adolescents who participated in online ERITA appreciated the support the therapists gave them, and the content was perceived as helpful [21]. In another study that targeted NSSI in an inpatient child and adolescent psychiatric setting, participants especially welcomed learning skills, and also appreciated self-management and self-care as well as communication and openness with the healthcare professional [22].

Treatments that address NSSI generally begin with an assessment of the behavior’s origin, development and maintenance in the context of previous and current life circumstances. The evidence-based treatment is based on the assessment. Examples of components in treatments that address NSSI generally include psychoeducation about NSSI and emotions, and teaching skills for emotion regulation, communication and problem-solving. Furthermore, the importance of reducing emotional imbalance and vulnerability is emphasized, as well as identifying the functions of NSSI, building a life worth living, learning how to use functionally equivalent behaviors with less long-term negative consequences, promoting help-seeking and social support, and targeting situations in the environment that trigger and maintain NSSI. Treatments should also focus on strengthening self-care, autonomy, and self-efficacy [23]. A clear structure for treatment is further recommended, together with an empathic, validating communication style based on respectful curiosity that encourages self-compassion [23, 24]. Shame is often embedded in the experience of NSSI. A special focus on the therapeutic relationship is necessary to avoid detrimental transactional processes between invalidating therapeutic responses and shame [25], for example, in individuals with NSSI who often struggle with self-criticism [26], which typically tends to exacerbate distress and NSSI. Most adolescents with NSSI do not, however, receive treatment. One barrier is individuals’ hesitation in disclosing NSSI [27, 28]. Other barriers are lack of knowledge and reluctance in mental healthcare professionals to treat NSSI [29].

Ambivalence to let go of NSSI is not unusual. NSSI is perceived as helpful, at least in the short term. Repetitive NSSI has in the literature also been conceptualized as a behavioral addiction with strong urges preceding the behavior, repetition, relapse, and continuation despite negative long-term consequences [30]. The process of NSSI cessation can therefore seldom be rushed [31], which can be challenging for healthcare professionals with insufficient knowledge of NSSI. Motivation for NSSI cessation thus often fluctuates and the process toward recovery is not linear [32], which needs to be considered in treatment.

Earlier research on how individuals experience healthcare and treatment have mainly focused on self-harm, irrespective of suicidal intent [27, 33]. Few studies have, however, focused specifically on NSSI in the adolescent population. Qualitative research focusing on adolescents’ experiences of healthcare and treatment for self-harm, irrespective of intent, has shown that patients being treated in the healthcare system have experienced stigma, negative attitudes, being judged, and not receiving adequate help. Furthermore, adolescents perceived that healthcare staff had preconceptions and lacked specific knowledge of self-harm. There have also been reports of healthcare staff using punitive measures [29, 34–36]. Previous research has shown that adolescents express a wish for a more collaborative relationship and open communication with healthcare professionals [37].

There are also reports of positive experiences of treatment and care. Individuals with self-harm, irrespective of intent, especially appreciated having someone to talk to and being taken seriously [29, 34, 35, 38]. Participants further appreciated continuity and predictability in the therapeutic relationship and in healthcare contacts. Being presented with a solution to the problem was not highlighted as the main objective for participants. Instead, a caring approach and being listened to were emphasized [29, 34–36, 38]. The need to consider general functioning, mood, health, quality of life and well-being rather than solely focusing on self-harm frequency has also been emphasized by those with lived experience of NSSI [34, 39].

Since many individuals still experience negative attitudes and stigma-inducing behaviors toward NSSI in healthcare, it is important to obtain a deeper understanding of the specific experiences of individuals with lived experiences to improve healthcare [21]. Few earlier studies have, however, focused on how individuals with NSSI experienced the psychiatric treatment and care they received when they were adolescents.

The aim of the current study was to explore young adults’ perceptions and lived experiences of the psychiatric treatment and care they received for NSSI during adolescence.

## Method

This qualitative study is part of a follow-up study of neurobiological markers of NSSI [40, 41]. The current qualitative study is presented according to the Consolidated criteria for reporting qualitative research (COREQ; [42]).

### Participants

Thirty adolescents with ongoing NSSI who were between 15 and 17 years (M = 15.9, SD = 0.8) were recruited from child and adolescent psychiatry (CAP) from June 2016 to March 2018 as part of a study examining neurobiological markers of NSSI [40, 41]. Inclusion criteria for participants were having engaged in five or more instances of NSSI during the past six months, independent of psychiatric diagnosis, and being a female between 15 and 18 years of age. Exclusion criteria were current or life-time diagnosis of schizophrenia, bipolar or psychotic disorder and/or alcohol/drug dependence, and IQ below 80. The most common psychiatric diagnoses were depression and attention deficit hyperactivity disorder (ADHD) or attention deficit disorder (ADD). About a third of the adolescents were on medication, most commonly selective serotonin reuptake inhibitors (SSRI)/selective noradrenaline reuptake inhibitors (SNRI). See Mayo et al. [40] and Perini et al. [41] for more information on the original study sample demographics.

In the five-year follow-up study, the 30 young adults were approached in 2021–2023. Of the original participants, one did not want to take part in the follow-up, one could not be reached, and two participants gave oral consent to participate in the first contact, but written informed consent was not obtained and they were thus not included in the study sample. In total, 26 young adults (86.7% of original sample, 100% assigned female sex at birth) between ages 20–22 years (*M* = 21.2, *SD* = 0.8) were included and interviewed.

Of these, 19 (73.1%) had ceased NSSI since more than one year ago, two (7.7%) had their latest NSSI episode 6–8 months ago, and five (19.2%) still had ongoing NSSI. At the time of the interview, 12 (46.2%) had ongoing contact with psychiatric services, one (3.8%) participant had contact with primary care and 11 (42.3%) did not have ongoing contact with psychiatry, and an additional two participants (7.7%) were waiting for assessment/treatment. Participants had different psychiatric diagnoses both separately and comorbidly. Of the 26 participants, four (15.4%) did not meet criteria for any psychiatric disorder, including unspecified. Twelve participants were on current medication (SSRI/SNRI, ADHD-medication, neuroleptics and stabilizers) and 14 had no medication at the time of the interviews. See Table 1 for participants’ demographics.

**Table 1:** Participant demographics, *N* = 26

Demographic characteristics	Total sample, n (%)
Sex	
Assigned female sex at birth	26 (100.0)
Age *m* (*sd*)	21.2 (0.8)
Healthcare contact	
Psychiatric contact	12 (46.2)
Primary care contact	1 (3.8)
Waiting for psychiatric assessment/treatment	2 (7.7)
No ongoing healthcare contact	11 (42.3)
Psychiatric diagnoses*	
Depression	7 (26.9)
Autism	7 (26.9)
Autistic traits	4 (15.4)
ADD/ADHD (including unspecified)	14 (53.8)
Eating disorder (including unspecified)	6 (23.1)
Bipolar disorder	2 (7.7)
Anxiety disorder (including unspecified)	10 (38.5)
PTSD	4 (15.4)
Borderline personality disorder, including traits	10 (38.5)
No diagnosis	4 (15.4)

*Note*. ADHD/ADD = Attention Deficit Hyperactivity Disorder/Attention Deficit Disorder, PTSD = Posttraumatic Stress Disorder. *Participants could have several diagnoses

### Data collection

Participants were first contacted by telephone with initial information about the follow-up study and written information was then sent by letter to participants. After having read through the written information, participants were again contacted by telephone and gave oral consent to participate. When participants came to the assessment and interview session at the hospital, written informed consent was obtained. For participants living far away, digital interview by skype/zoom was offered as an option. For the informant who chose this option, the signed informed consent was sent in by mail beforehand. One participant was interviewed digitally, and 25 participants were interviewed face-to-face. In addition to the interview, self-report questionnaires were filled in and psychiatric symptoms and diagnoses were assessed. The interview came first in the session, and the questionnaires and assessment were completed afterwards. The interviews took place from November 2021 to February 2023.

### Interview

Interviews were conducted by two female licensed psychologists (HA, MZ) with experience of clinical work and research about NSSI and clinical child and adolescent psychiatry. One of the interviewers (MZ) had been involved in the assessment procedure of the original study five years ago, and had met the participants in this role, but did not have any ongoing clinical relationship to the participants. The interviewers and the aim of the study were presented at the beginning of the interview. The interviews were semi-structured with open questions that focused on three areas: the process of NSSI cessation; the benefits and barriers of NSSI related to NSSI cessation; and participants’ experiences of healthcare and treatment of NSSI. Prompts were used when needed to obtain more details regarding the participants’ experiences. Mean length of interviews was 25 min (varying between 10 and 40 min). Interviews were recorded and transcribed verbatim.

### Ethical considerations

The study was approved by the Regional Ethical Review Board of Linköping and the Swedish Ethical Review Authority (2015/273 − 31; 2016/224 − 32; 2021–04328). All participants in the study signed an informed consent form. Personal identifiable information has been anonymized and removed, and names have been replaced.

### Analysis

Data were analyzed with thematic analysis (TA) according to Braun and Clarke [43, 44] with an essentialist/realist approach underpinning the analysis. The main aim was to capture the participants’ experiences of the treatment and care they received five years ago. Thus, the data were analyzed inductively, based on data, and on a semantic/explicit level to keep close to the participants’ expressions. The analysis was made in the six steps described in TA. The interviews were read several times to become familiar with the material. Codes were initially produced by AM and ES, and themes were created and discussed together with all authors. AM, ES, HA and MZ reviewed different ways of clustering the codes several times until consensus was reached on which themes best covered the interpretation of the data. The final themes were reviewed by RH. All authors labelled the themes, and the results were approved by all authors. The themes were reviewed several times, and the analysis had a continuous movement back and forth between the six steps in TA [43, 44] and between the transcripts, the codes, and the themes. This meant reading, coding, and finding themes, but also modifying codes and themes after checking if the themes were compatible with the codes and transcripts.

## Results

The analysis resulted in three overarching themes and five sub-themes. See Table 2. The first theme, Changed perceptions in retrospect, summarizes participants’ view of how their attitudes and perception of themselves and the treatment changed over time. The next theme, The importance of a collaborative conceptualization, includes three subthemes that together describe participants’ experience of treatment, and whether they were actively invited to engage in the conceptualization of NSSI and focus of treatment. The last theme, Lasting impression of the relationship, contained two sub-themes that reflect different descriptions of the relationship with the healthcare professional.

**Table 2:** Main themes and sub-themes

Main themes	Sub-themes
Changed perceptions in retrospect	
The importance of a collaborative conceptualization	Agreement and disagreement - continuum Lack of clarity Vague memories
Lasting impression of the relationship	Validation Stability and trust

### Changed perceptions in retrospect

Participants described a change in how they viewed themselves and their treatment when they looked back at when they were adolescents engaging in NSSI. Most of the participants expressed having negative perceptions of themselves and their live situation five years ago, but since then they had experienced a change in perspective. For instance, one participant (Charlotte) expressed not being able to see the impact and help the treatment had at the time, but today perceived the treatment as helpful. Another participant, Marie expressed:

I remember being so incredibly angry… I got so provoked, I was so angry all the time… I was very angry at healthcare as well […] because when you’re in it you only think that I go to a psychologist and now that psychologist should solve all my problems, and that’s not how it works.

Marie described how her view of psychological treatment had changed with an increased understanding and a new perspective. This change of perceptions over time was found in most of the participants’ experiences, and this theme was interpreted as having an undertone of maturation. Some of the participants experienced feeling alienated from others as adolescents and thought that no one could understand them. The experience of feeling misunderstood was also related to treatment.

I sat there and said “yeah mm I can try” but I knew that it wouldn’t work [...] whatever you say right now, it won’t work because I know how I am, regardless of what you say, like think about this or think about going out with your dog or whatever, but you’re not there, you’re not there in your head. (Melissa)

Melissa did not believe that treatment could help her at the time and experienced not being understood by others, and not being ready for proposed changes. In addition to treatment, participants also expressed changed perceptions in how they perceived themselves.

I experienced that I did some things, at least temporarily [...] also maybe because I had decided that I wanted to feel bad a while and then the behavior [NSSI] fitted in the lifestyle that I wished to live then. (Anna)

Anna described a greater understanding of the function of her NSSI in retrospect. The participants expressed different reasons for why they had ceased with NSSI, but also expressed not being able to see those reasons back then. Participants described being very self-critical as adolescents, which also changed with time. Together, the experiences of a change in perspective with a greater understanding of themselves, their situation and their treatment over time, were conceptualized as the overarching theme Changed perceptions in retrospect.

### The importance of a collaborative conceptualization

The overarching theme The importance of a collaborative conceptualization describes the participants’ experiences of the focus of the treatment they received. This theme captures how the conceptualization of NSSI affected how treatment was perceived. It further reflects the importance of mutual collaboration between the healthcare staff and participants, and the degree to which they were in a productive alliance when addressing NSSI. It consisted of three subthemes: Agreement and disagreement - continuum, Lack of clarity and Vague memories.

#### ***Agreement and disagreement - continuum***

This subtheme summarizes participants’ experiences of whether or not they were in agreement concerning the focus of treatment, and describes the degree to which participants were included in the conceptualization of NSSI and planning of treatment goals. What unified the positive experiences of the treatment was understanding the treatment rationale and a clear and jointly agreed-upon focus, where participants were invited to participate. For instance, working together with the therapist using chain analysis or functional analysis to jointly increase the understanding of the processes leading up to and maintaining NSSI was viewed as helpful by several participants, which also created a sense of collaborative exploration and joint conceptualization of the problem, something which was interpreted as a key for satisfaction.

For me it was helpful with these chains, that I always, but why, what happened, what triggered my self-injury. It felt good to be able to look back, talk about my situation, what it looked like, what I thought…I describe which feeling, when you looked back you perhaps realized that it was something completely different. (Kim)

In contrast, some participants experienced that the treatment focus was off target. This was interpreted as a disagreement between the participant and therapist, which led to a sense of dissatisfaction. For instance, whether NSSI was conceptualized by the therapist as a symptom in its own right or as an expression of other problems, and consequently, if NSSI should be the focus of treatment or not, was one area of potential disagreement. This disagreement was, however, seldom verbalized explicitly between the adolescent and mental health professional.

No, it [NSSI] was barely in focus really. It was a lot of focus on me needing to process everything, like how I felt, what I had done… […] it felt like the only time NSSI was brought up was in the research study. It wasn’t at the meetings, and it was like it was some side thing that you might take care of later. So… then I quit everything I was like, Ok… I don’t want to come back here because I just feel bad here. But a part of me was disappointed that they didn’t even try… like, why? (Jamie)

Jamie experienced the lack of focus on NSSI as disappointing. Other participants, on the other hand, didn’t appreciate too much focus on NSSI, and preferred focusing more on underlying aspects, like Sara in the excerpt below.

I want more that we forget about the self-injury and think that self-injury is like coughing when you have pneumonia […] you don’t want medicine because you shouldn’t be coughing because it is so hard for other people seeing you cough, but it is to cure the disease that makes you cough. (Sara)

Taken together, the participants described various experiences relating to how well the focus and conceptualization of the treatment was discussed jointly and emphasized the importance of a clear and agreed-upon focus. Whether NSSI should be in focus or not, however, could differ depending on participants’ experiences and wishes.

#### ***Lack of clarity***

Some participants expressed difficulties recollecting what the focus of the treatment was. The treatment was described as vague and without direction, which was conceptualized as a Lack of clarity. The participants didn’t express direct dissatisfaction with the treatment, but rather experienced a lack of a treatment plan. Hannah expressed “It felt like nobody knew like why I was there? […] they didn’t, and I didn’t, we never had like a main focus…it was more like the meeting of the day.” A lack of understanding of the treatment they received was further described by the participants. Sometimes this was a lack of interventions for NSSI, other times the interventions were confusing, lacked direction or were incongruent. For instance, several participants experienced receiving sporadic tips about how to handle their NSSI without a solid rationale or long-term plan. Anna said: “I don’t actually think that I received that much treatment for it… maybe they gave the usual tips, put an ice cube.”

#### ***Vague memories***

Several participants expressed difficulties remembering the time of their NSSI and the content of the treatment and referred to the adolescent years with mental health problems as a blur. For instance, Amanda said “I don’t even remember how I was treated. I don’t remember if I got any help for my self-injury.” Some experienced a general loss of memory, whilst others experienced the memories as incongruent.

### Lasting impressions of the relationship

The relationship with the healthcare professional was interpreted as a core component of the treatment and the impressions were salient several years later. Participants expressed the importance of being understood, taken seriously and not being judged. A relationship that included validation, stability, and trust was a prerequisite for experiencing the treatment as positive. These experiences were conceptualized as Lasting impressions of the relationship and consisted of the subthemes Validation and Stability and trust.

#### ***Validation***

The experiences of the relationship to the healthcare professionals varied, mainly depending on whether the participant had felt validated or not. Several participants remembered being treated well by the healthcare professionals, and reported being listened to, accepted, and understood.

I wasn’t embarrassed to talk to them about it, so I was treated really well… Always understanding, they gave me tips. Yeah, like they were good, they didn’t do anything wrong, they treated me really well […] I could be myself. No one judged me… (Jessica).

In contrast, some participants had more negative experiences from the interaction with healthcare professionals. Their expressions were interpreted as experiences of being invalidated. Some participants felt questioned by the healthcare professionals when they were asked why they self-injured. Other participants had experienced feeling invalidated when healthcare professionals commented on their wounds. Maxine expressed her reaction to healthcare professionals playing down her NSSI.

“Only superficial cutting”, adding the word “only” can mean that it is evaluated as not being, like, for real.

Some participants remembered experiencing impulses to turn up the volume of their NSSI in order to be taken seriously, in situations in which NSSI was minimized and/or dismissed by staff. How and when strategies for replacing NSSI were communicated often resulted in participants feeling misunderstood, and that the extent of their mental health struggles was not fully appreciated. It was not the strategies per se that were experienced as invalidating, but rather how they were communicated. Too much focus on quickly getting rid of the behavior was experienced as minimizing the complexity of NSSI. Some participants experienced being judged by the healthcare professionals, but the participants often found it difficult to concretize the specific thing the healthcare professionals said or did. The experience of invalidation varied, but the experiences of validation and invalidation made a strong impression and were salient several years later.

#### ***Stability and trust***

Several participants expressed the importance of time and predictability to trust the healthcare professional enough to disclose and open up about NSSI.

It takes a very long time to open up, and I know that it has happened that I got the question [about NSSI] in a meeting, but I said no then. Because…either I don’t feel comfortable, or so, maybe I’m stressed and don’t want to talk about it. (Cornelia)

Sara described the consequences of not knowing how long she would receive treatment, perceiving a need to prove that she needed the treatment: “I believe it almost creates an inner competition… that you always have to prove how bad you’re feeling in a physical way even in healthcare.” Lack of stability and trust created uncertainty in some of the participants, who said that a safe and stable relationship was helpful in addressing NSSI in treatment.

## Discussion

This study explored how 26 young adults with lived experience of NSSI perceived the treatment they received as adolescents. Most participants no longer engaged in NSSI at the time of the interviews. The interviews were analyzed with thematic analysis and resulted in three overarching themes: Changed perceptions in retrospect, The Importance of a Collaborative Conceptualization, and Lasting impression of the relationship. In general, participants experienced a change in perspective of themselves and the treatment, with a deeper understanding of the processes over time. Further, the importance of agreeing and being included in treatment planning was emphasized. The relationship to the healthcare professional and the way in which the healthcare professional talked about NSSI made a strong impression and was salient several years later.

### Changed perceptions in retrospect

The first theme pinpointed participants’ change of perspective with the passing of time, when looking in the rear-view mirror at their experiences as adolescents. The change reflected in this theme covered processes related to themselves, their NSSI and previous life context, as well as the treatment. Several participants reported that during adolescence, when they were still actively engaging in NSSI, they had negative perceptions of themselves and the treatment, but that they had gained a more nuanced perspective with time.

Participants mentioned being very self-critical during their adolescent years, and also experienced anger and criticism toward healthcare, doubting both their own and healthcare’s ability to deal with their life situation. It is known that self-criticism is associated with NSSI [26], and self-punishment is a commonly endorsed function of NSSI [10]. That NSSI decreased with age is in line with earlier research [3]. In the current study, participants reported that the negative perspective of self also decreased as they got older and were no longer actively engaging in NSSI. Participants reflected on how their own developmental processes during adolescence and mind-set at the time influenced how susceptible they were to interventions. Some participants also touched on issues related to identity formation, which is a critical developmental task during adolescence. Gandhi et al. [45], for example, previously showed a bidirectional relationship between NSSI and identity in a longitudinal study of adolescents, with identity confusion predicting NSSI and vice versa. An earlier systematic review [27] found that individuals with NSSI were in an ongoing process of constructing and negotiating their identity as patients with NSSI. Results from the current study also confirm that this process is ongoing and active from adolescence to young adulthood. Except for one previous study [34], where individuals were interviewed about their experiences of inpatient child and psychiatric healthcare 15 years earlier, most other qualitative studies that explore participants’ experience of healthcare in relation to self-injury do so in closer proximity to the healthcare contact. Our study encompasses the aspect of time, which makes it possible to understand changes of perspective that come with time.

Some participants reported that their motivation was lacking or fluctuating when they were in treatment. This is not uncommon in NSSI cessation processes [31], and NSSI cessation is best understood as a non-linear process with setbacks and fluctuating motivation [32]. NSSI serves several functions [10] and helps individuals to cope with life stress and challenges, which need to be considered when treating NSSI [31]. Several participants in our study could give several reasons to cease NSSI, which they could not do earlier, and described what Vansteenkiste et al. [31] refer to as a lack of internal motivation during the adolescent years.

It is not uncommon that people other than the person with lived experience of NSSI, such as parents, teachers, and clinicians, argue that NSSI needs to stop immediately and therefore focus on problem-solving, emphasizing change at the expense of understanding, validation and acceptance [32]. It is often difficult for parents and healthcare professionals not to force change toward cessation, since NSSI most often is conceptualized as a dangerous and potentially life-threatening behavior [31]. Individuals with NSSI thus often experience external pressure and control from others to cease NSSI, which can restrict autonomy and ultimately aggravate NSSI [46].

### The importance of a collaborative conceptualization

The three sub-themes under the main theme interpreted as The Importance of a Collaborative Conceptualization all relate to the experience of treatment. The common thread was the agreement-disagreement continuum which was conceptualized as a central part of how the treatment was perceived. Participants who had experience of mental health professionals or therapists who overtly expressed and included participants in the conceptualization of NSSI, and where the goals and focus were agreed upon, were most satisfied with the treatment content. Participants that had taken part in a structured treatment, such as DBT or emotion regulation skills training, were generally more positive. Specific aspects that were appreciated were focusing on emotions, chain analyses of emotions and NSSI and strategies for regulating emotions and coping with NSSI. These are modules that are recommended and incorporated in modern treatments of NSSI [15, 16].

In cases where therapists and participants had different views, mainly on whether NSSI should be targeted in its own right or be viewed as a symptom of some underlying issue or problem, and when this disagreement was not negotiated and discussed explicitly, participants were more negative to the content of treatment. Such lack of communication has been problematized in treatment of self-harm, irrespective of intent, in an earlier systematic review [33], despite adolescents specifically requesting collaboration and open communication [37]. Participants’ preferences for focus differed: some wanted more explicit focus on NSSI, whereas others sought more focus on underlying mental health concerns or life circumstances. The common theme was that treatment focus needed to be discussed and agreed upon jointly, and a lack thereof influenced perception of healthcare.

Agreement on treatment goals and tasks in an atmosphere of collaboration is the ground for a constructive treatment alliance [47]. When patients in general describe what is important for them, shared directionality is a recurrent issue [48]. It is important that the goals are personal [49], and that therapist and patient together identify and clarify obstacles for change [50]. In order to achieve agreement, goals and tasks need to be negotiated. Therapist sensitivity about patients’ unformulated or vague goals and expectations is essential [51].

Earlier qualitative work [39] emphasizes the need to broaden the perspective to include overall health and functioning, and that too much focus on NSSI as a marker of distress is neither appreciated nor meaningful. In the current study, some participants confirmed this perspective whilst others wanted to focus on NSSI and experienced that NSSI was left out of treatment. In clinical samples, NSSI occurs together with different diagnoses [6] and serves several functions [9, 10]. Given the heterogeneity of the NSSI population, it is reasonable that needs and preferences differ, but open communication and joint decision-making on what to target were emphasized and improved perceptions of treatment content.

Several participants described not remembering if and what treatment they had received and that the adolescent years with mental health problems and NSSI were a blur. There are no robust findings of differences in working memory in participants with NSSI compared to healthy controls [52, 53]. Common comorbid conditions such as neurodevelopmental disorders, trauma or depression are, however, known to impact attention and memory [54, 55].

### Lasting impression of the relationship

In addition to being included in the conceptualization of NSSI and jointly agreeing on the focus and goals of treatment, the relationship to the mental health professional was another important factor that influenced how treatment was perceived by participants. Participants’ recollection of encounters with healthcare professionals’ attitudes and communication style was still highly salient, even though several years had passed.

The general importance of the therapeutic relationship in treatment and psychotherapy research has been consistently found in earlier research [56, 57], and also specifically in relation to NSSI [27, 35, 36, 38]. Studies of alliance building underline that confidence in the treatment as well as confidence in the therapist are basic aspects of a constructive therapeutic relationship [58, 59].

In the current study, positive experiences were identified, such as being listened to, taken seriously, understood, and not judged, together with negative experiences of being invalidated and misunderstood. Participants in earlier studies have emphasized the impact of the human contact in treating self-harm, irrespective of intent, and that practitioners generally underestimate the importance of the relationship [29].

Applying a problem-solving perspective too early in the treatment process was not uncommon and was mainly perceived as invalidating by participants. This included an extensive focus on getting rid of NSSI and receiving suggestions for replacing the behavior with other less harmful behaviors, such as drawing on skin. Such so-called harm-minimizing strategies, mainly sensation or process proxies, using elastic bands or drawing on skin have little empirical support, and are also mostly perceived by young people with self-harm to be ineffective [60], and thus need to be used with caution. Other non-optimal clinician approaches were minimizing and dismissive comments. This is not uncommon and confirms earlier studies of self-harm, irrespective of intent [29]. Also, too much focus on NSSI frequency as a proxy for well-being, resulted in participants feeling misunderstood. Frequency of NSSI does not necessarily correlate with mental health or need of help [39]. Having the injuries labeled with judgmental language, such as “that wasn’t that bad”, was perceived as hurtful and risks individuals needing to turn up the volume or frequency of NSSI to be taken seriously [27, 39].

Participants also emphasized the role of shame in relation to NSSI and that they needed time to build trust to disclose NSSI, which was not always possible in the CAP setting. Challenges related to inadequate number of sessions and abrupt termination of treatment have been described earlier in relation to self-harm, irrespective of intent [35]. Also, confidentiality, and breach of confidentiality in relation to NSSI, needs special consideration and is a recurrent issue for adolescents, both in the current study of NSSI and self-harm, irrespective of intent [38].

### Limitations

All participants were assigned female sex at birth. More research of healthcare experiences is needed with males and ethnically diverse samples. Every participant had experiences from CAP and it is this treatment that is referred to in the current study. Results are thus not transferable to males or all treatment settings. A majority of participants were no longer engaging in NSSI. Furthermore, they were in different phases toward recovery of NSSI and other mental health problems, which potentially could influence how earlier treatment during adolescence was perceived. The background of the researchers involved could potentially influence the analysis. Interviews were conducted by two licensed female psychologists (HA, MZ) with experience of CAP and treating NSSI. Analyses were conducted by three females (HA, ES, MZ) and two males (RH, AM), with varying ages and experience of clinical work and research, however, which contributes to minimizing bias in interpretation of data.

### Clinical implications

The lived experiences of participants with NSSI who have been in contact with CAP during adolescence have several important implications for clinicians. It is important to embark on the journey of assessment and treatment of NSSI collaboratively and include the perspective of the adolescent. The conceptualization of NSSI, and agreement on focus, goals and methods that guide treatment, need to be negotiated openly together. It is striking how seldom adolescents expressed their dissatisfaction directly to the healthcare professional. Staff therefore need to be mindful of this and initiate ongoing discussions about adolescents’ thoughts and feelings related to the treatment and the relationship. It is also important that they become aware of and address conflicts and ruptures in the treatment alliance. Such attention may not only prevent premature termination, but also has the potential to improve adolescents’ competence for interpersonal conflicts [61]. The actual focus and content can differ and should be individualized. Furthermore, adolescents need clarity in aspects such as treatment length and plans for termination, where continuity is preferred. Clarity concerning breach of secrecy, such as when parents need to be contacted, is also essential.

It is also important that clinicians take into account that adolescents with NSSI in a clinical setting might be suffering from impaired attention and memory, and therefore clinicians need to also rely on written material and summarize therapeutic work in writing.

Furthermore, relationship variables such as kindness, warmth, empathy and validation, and a non-stigmatizing communication style are crucial components and have a large impact on how treatment is perceived. Being too quick to problem-solve, and excessively focusing on the need to stop NSSI can potentially communicate that the problem is less complex than it actually is and can therefore be perceived as invalidating and dismissive. Also, minimizing comments such as “that wasn’t too bad” were not perceived as helpful, and a more descriptive language is preferred. Knowledge about the complexity of NSSI is needed for professionals that come in contact with NSSI. It is often more effective to promote internal motivation to change based on understanding, validation, and empathy and to avoid limit-setting, rules, and bans. Cessation processes can seldom be rushed and take time.

## Conclusions

In this study, 26 young adults with NSSI looked back on the treatment they received as adolescents for NSSI. Participants view of themselves and the transaction between themselves and the health care professional changed with time. This change of perspective was a major theme that was developed in the analyses. Furthermore, the importance of an agreed-upon structure and goals, content, and focus, which need to include the perspective of the individuals with lived experience of NSSI in an openly communicated conceptualization, was emphasized. A lack of agreement, often unspoken, resulted in a negative experience of healthcare treatment. Finally, the relationship to the healthcare professional was important and salient to participants several years later. Aspects such as stability and trust, and being taken seriously, were emphasized.

## Data Availability

Data is not available since we do not have participants’ or ethical permission to share data.
